# Differences in the Clinical Manifestations and Host Immune Responses to SARS-CoV-2 Variants in Children Compared to Adults

**DOI:** 10.3390/jcm13010128

**Published:** 2023-12-26

**Authors:** Salih Demirhan, David L. Goldman, Betsy C. Herold

**Affiliations:** Department of Pediatrics, Division of Infectious Diseases, Albert Einstein College of Medicine, The Children’s Hospital at Montefiore, 1225 Morris Park Avenue, Bronx, NY 10461, USA; sdemirhan@montefiore.org (S.D.); dagoldma@montefiore.org (D.L.G.)

**Keywords:** pediatric COVID-19, SARS-CoV-2 variants, innate immunity

## Abstract

The COVID-19 pandemic challenged the medical field to rapidly identify and implement new approaches to the diagnosis, treatment and prevention of SARS-CoV-2 infections. The scientific community also needed to rapidly initiate basic, translational, clinical and epidemiological studies to understand the pathophysiology of this new family of viruses, which continues to evolve with the emergence of new genetic variants. One of the earliest clinical observations that provided a framework for the research was the finding that, in contrast to most other respiratory viruses, children developed less severe acute and post-acute disease compared to adults. Although the clinical manifestations of SARS-CoV-2 infection changed with each new wave of the pandemic, which was dominated by evolving viral variants, the differences in severity between children and adults persisted. Comparative immunologic studies have shown that children mount a more vigorous local innate response characterized by the activation of interferon pathways and recruitment of innate cells to the mucosa, which may mitigate against the hyperinflammatory adaptive response and systemic cytokine release that likely contributed to more severe outcomes including acute respiratory distress syndrome in adults. In this review, the clinical manifestations and immunologic responses in children during the different waves of COVID-19 are discussed.

## 1. Introduction

One of the most striking observations from the beginning of the SARS-CoV-2 pandemic was the marked difference in disease severity in children compared to adults. Al-though infection rates were similar or even higher in children, most children were asymptomatic or had mild illness and rarely required hospitalization. In contrast, the morbidity and mortality in adults was substantial. Approximately, 15.6 million US children were infected with SARS-CoV-2 from March 2020 to May 2023, which accounts for ~17.9% of total cumulated cases; notably, children (<18 years) comprise ~22% of the total US population [1]. Thus, the age-related differences in disease severity are not attributable to reduced susceptibility but more likely reflect differences in host responses. Moreover, the rates of infection and clinical manifestations changed with the emergence of new variants that differed in tissue and cell tropism and the ability to elicit and evade host immune responses. Natural and vaccine-induced immunity, treatment strategies and changes in social distancing policies also impacted the clinical manifestations and host responses to new viral variants. This review focuses on how children responded to successive waves and why they continue to experience less morbidity and mortality than adults.

## 2. Clinical Manifestations of Acute COVID-19 in Children with Successive SARS-CoV-2 Waves

During the first wave of SARS-CoV-2 infection (March–November 2020), the ancestral virus originating from Wuhan, China was rapidly replaced by the first major variant of concern, the Alpha variant (lineage B.1.1.7), which carried multiple mutations including a major change in the spike protein (D614G) [2]. The most common symptoms reported in infants and children were fever (46.3%), cough (36.9%) and less frequently, dyspnea (6.5%) [3]. Gastrointestinal symptoms including diarrhea, vomiting and abdominal pain were also documented. Hospitalizations among children, however, were uncommon.

**Figure 1:**
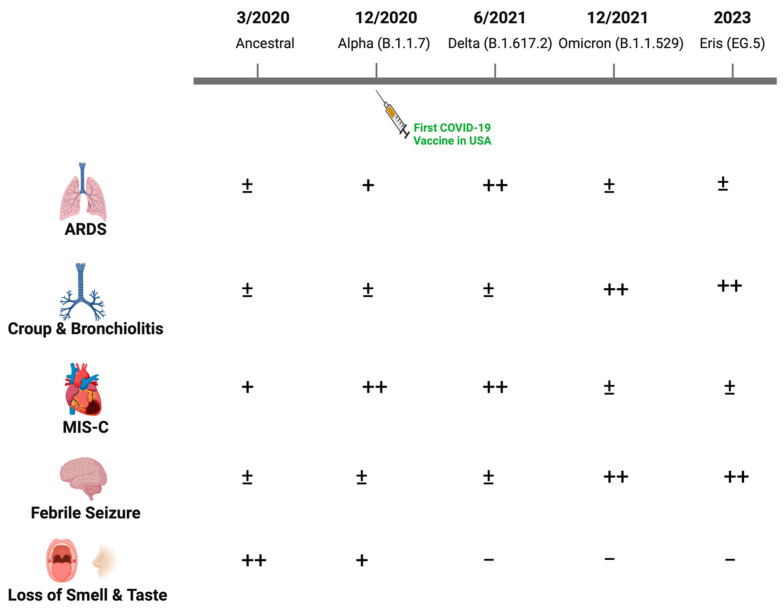
Changes in the clinical manifestations of SARS-CoV-2 in children with evolving viral variants. While most children infected with SARS-CoV-2 have been asymptomatic or developed self-limited fever with mild respiratory or gastrointestinal symptoms throughout the pandemic, the relative frequency of other clinical manifestations changed with emergence of new dominant viral variants as illustrated (−, ±, +, ++).

Clinical manifestations were similar during the second major wave through the fall of 2021, when the Delta variant (B.1.617.2) dominated. However, the Delta variant was associated with greater disease severity and increased hospitalization rates, possibly reflecting increased transmission, enhanced viral replication and immune escape [4]. Older children and adolescents with risk factors similar to those observed in adults including obesity progressed to acute respiratory distress syndrome (ARDS), although the incidence was low relative to what was observed among adults. The disease spectrum changed substantially with the emergence of Omicron variants (primarily B1.1.529) in December 2021 (Figure 1). More upper rather than lower respiratory tract diseases, including bronchiolitis and laryngotracheobronchitis (croup), were described with Omicron. Loss of taste, which was relatively uncommon in children compared to adults in the earlier waves, was almost never reported with Omicron [5,6,7]. A single-center study from New York reported an increase in the proportion of pediatric patients hospitalized with croup from 1.1% pre-Omicron to 6.6% during the Omicron wave [8]. Similar findings were observed using the US National COVID-19 Cohort Collaborative, which found that upper airway infection rates associated with SARS-CoV-2 disease increased from 1.5% to 4.1% from the pre-Omicron to Omicron period [9]. There was also an increase in febrile seizures associated with SARS-CoV-2 infections in the Omicron period, although the underlying reasons for this increase are unclear [10,11,12].

The emergence of Omicron variants resulted not only in changes in clinical manifestations but also in hospitalization patterns [12,13,14,15]. While older children and adolescents with comorbidities similar to those identified in adults including obesity and diabetes dominated pediatric hospitalizations pre-Omicron, this shifted to younger children with the emergence of Omicron variants. For example, the Center for Disease Control (CDC) surveillance data available from March 2020 to Feb 2022 found that hospitalization rates for children ≤4 years old were five times higher with the Omicron compared to the Delta surge [13]. Nearly half of these hospitalizations (*n* = 2562) were infants younger than 6 months of age and the majority had no underlying comorbidities [13]. Although admission rates were higher, length of stay and intensive care unit (ICU) admissions were lower. For example, Omicron was associated with significantly lower odds of moderate or severe disease compared to the Delta variant (adjusted odds ratio: 0.12) [16]. The clinical spectrum of disease with more recent Omicron subvariants, including XBB.1.16 (Arcturus) and E.G.5 (Eris), which have replaced the original Omicron variant, are not yet fully defined. However, early reports from India, where this variant emerged in April 2023, noted an increase in rates of infections in younger infants compared to older children (37.4% vs. 13.3%; *p* < 0.001). Mild respiratory symptoms and non-purulent conjunctivitis were the primary manifestations described in children [17].

The difference in symptoms and disease severity (greater with Delta), and the predilection for upper versus lower respiratory tract disease (more upper tract disease with Omicron variants) may be attributed, in part, to mutations in the spike protein that allow for differential tropism, entry and spread [18]. The SARS-CoV-2 spike protein is cleaved into two subunits, S1 and S2. The S1 subunit binds to angiotensin-converting enzyme-2 (ACE2), allowing for the exposure of the S2′ site. If the cell expresses transmembrane protease serine 2 (TMPRSS2) and the spike protein has affinity for this enzyme, the S2′ site is further cleaved at the plasma membrane, which promotes viral entry by direct fusion. If TMPRSS2 expression is limited and/or there is reduced affinity for the enzyme, the virus is internalized by endocytosis and S2′ is cleaved by cathepsin L within the endosomal compartment. Lower tract lung alveoli express higher levels of TMPRSS2, whereas cells in the upper respiratory tract express higher levels of cathepsin [19]. Moreover, Omicron variant spike proteins are less efficiently cleaved by TMPRSS2 compared with those of the Delta variant. This results in greater dependence on entry via endocytosis and reduced ability to infect lung cells, as evidenced by studies with pseudotype viruses [20]. These differences in cell tropism and entry mechanisms likely contribute to the differences in disease manifestations, with Delta causing more lung disease, whereas Omicron and its variants exhibit more upper respiratory tract disease. The TMPRSS2-mediated membrane fusion also promotes syncytia formation, which is associated with more severe lower tract disease. This phenotype was most pronounced with the Delta variant in which the P681R mutation in the spike protein enhanced fusogenicity and likely contributed to the increased pathogenicity [21].

## 3. Multisystem Inflammatory Syndrome

Another important clinical manifestation of SARS-CoV-2 that was relatively uncommon but unique to the pediatric population was the emergence of an acute post-viral syndrome designated as multisystem inflammatory syndrome in children (MIS-C). MIS-C was first described in England in April 2020 during the first COVID-19 wave and shared features with atypical Kawasaki disease or toxic shock syndrome. The syndrome is defined by the CDC using the following criteria: age < 21 years, evidence of recent (within 60 days) SARS-CoV-2 infection or exposure, fever, severity requiring hospitalization, laboratory evidence of inflammation and involvement of two or more systems (shock, cardiac, hematologic, gastrointestinal or dermatologic) without plausible alternative diagnoses. MIS-C may be associated with significant morbidity. A recent study found that over half of the patients had myocardial injury during the acute illness (37/69) and this persisted in 26/47 of the children 6–8 weeks later [22]. Notably, the incidence of MIS-C rapidly decreased with the emergence of Omicron and is now rarely reported [23,24].

The reasons why MIS-C is such a rare manifestation occurring, according to the CDC, in an estimated 1:3000 to 1:4000 children infected with SARS-CoV-2 and the pathogenesis of this syndrome have not yet been determined. Hypotheses include a hyperinflammatory cytokine storm response to the virus, induction of autoantibodies, and/or a superantigen response to a motif within S1 or to a non-SARS-CoV-2 antigen that becomes exposed perhaps because of a leaky gut [25]. Superantigens bind with high affinity to class II MHC outside the antigen binding groove and in the absence of antigen processing, cause polyclonal T cell activation and cytokine responses. Other studies have suggested that MIS-C may reflect more exuberant innate immune responses. This latter notion is supported by an epigenomics analyses of plasma cell-free DNA (cfDNA) that compared healthy children and children with acute COVID-19 to those with MIS-C and found higher levels of cfDNA originating from innate immune cells in the MIS-C patients, but no differences in cfDNA from adaptive immune cells [26]. In support of a role for adaptive immune responses, a different study comparing peripheral blood immune responses in hospitalized SARS-CoV-2-infected pediatric patients and those with MIS-C found that MIS-C was associated with greater T cell activation, including CX3CR1+ CD8+ T cells, which decreased with clinical recovery, suggesting a role for activated CD8+ T cells in the disease pathogenesis [27]. Another study showed stronger SARS-CoV-2-specific T cell responses (TNF and IFN-γ) in children with MIS-C compared to children with uncomplicated SARS-CoV-2 infection [28]. Genetic studies have suggested that there may be a predisposition to both MIS-C and to more severe COVID-19 disease. Single nucleotide polymorphisms in ACE2 rs2074192 (allele T), IFNAR2 rs2236757 (allele A), OAS1 rs10774671 (allele A), CD40 rs4813003 (allele C) and CASP3 rs113420705 (allele C) were identified in one study [29]. However, larger studies are needed to confirm these observations.

The incidence of MIS-C has declined with successive new waves [30]. In addition, the risk of MIS-C is much higher post-infection compared to post-vaccination [31]. These observations are consistent with several of the proposed mechanisms underlying the pathogenesis of MIS-C. Mutations in viral proteins and immune responses elicited by infection, vaccination or a combination of infection and vaccination (e.g., hybrid immunity) mitigate the hyperinflammatory response to the virus and may also reduce the likelihood of superantigen or autoantibody responses. In support of a protective role for vaccine immunity against MIS-C, a meta-analysis found that the pooled odds ratio for MIS-C in vaccinated compared to unvaccinated children was 0.04 (95% confidence interval: 0.03–0.06) [31]. Moreover, a recent CDC study of children with MIS-C showed a 23% higher rate of ICU admission for unvaccinated compared to vaccinated children (2 doses) [32].

## 4. Viral Coinfections

An additional factor that may contribute to the change in clinical symptoms associated with successive waves of SARS-CoV-2 infection is the role of respiratory viral coinfections. Early in the pandemic, coinfections with other respiratory viruses were rarely observed [33]. This may have resulted from isolation precautions, masks and related social behaviors, as well as the phenomenon of viral interference. Viral interference or exclusion refers to the ability of a virus to activate antiviral defenses that reduce coinfection with a second pathogen. This mechanism has been hypothesized to have contributed to the disruption of the 2009 influenza outbreak in Europe by rhinovirus [34]. Using an in vitro organoid model, rhinovirus infection before SARS-CoV-2 enhanced the interferon-stimulated gene responses and inhibited SARS-CoV-2 replication [35]. Similarly, influenza A induced a robust interferon response that suppressed SARS-CoV-2 replication in a human airway epithelial culture model, whereas SARS-CoV-2 did not enhance host cell defense during influenza coinfection or suppress influenza viral replication. Treatment with oseltamivir blocked influenza replication and reduced the associated innate response with a concomitant increase in SARS-CoV-2 replication. These in vitro findings suggest that the re-emergence of other viral infections may protect against SARS-CoV-2, although the interactions are undoubtedly complex and will require future study [36].

As expected with the relaxation of isolation behaviors, there was an increase in the incidence of respiratory viral coinfections. The US COVID-19-Associated Hospitalization Surveillance Network (COVID-NET) identified 4372 children hospitalized between March 2020 and February 2022 with SARS-CoV-2 infection; 62% were tested for other respiratory viruses. Coinfections were identified in 21% and were more likely to be detected in children who were <5 years of age, received oxygen support and were admitted to the ICU. SARS-CoV-2 coinfections with rhinovirus/enterovirus or RSV were each significantly associated with severe illness in younger children [37]. However, coinfections occurred at rates less than would be expected based on overall prevalence, suggesting that a viral exclusionary effect, albeit incomplete, between SARS-CoV-2 and other seasonal respiratory viral infections persists [38].

## 5. Post-Acute Sequelae of COVID-19

Distinct from MIS-C, which is an immediate post-SARS-CoV-2 syndrome, is a spectrum of symptoms of unclear etiology referred to as long COVID or post-acute sequelae of SARS-CoV-2 infection (PASC). In general, long COVID appears to occur less frequently in children than in adults. However, studies aimed at defining the incidence and etiology in children (and adults) have been complicated by the lack of objective findings, heterogeneous symptoms, absence of a specific diagnostic test and relatively small studies that often lack appropriate control populations and are often without controls. Moreover, only recently has there been a WHO consensus definition for pediatric long COVID (persistence or development of new symptoms 3 months after an initial SARS-CoV-2 infection lasting for at least 2 months that impact everyday function without other plausible etiology) [39]. The CLoCk study, a national cohort survey study being conducted in the UK, is addressing many of the limitations of earlier studies. The study is recruiting participants aged 11–17 years who tested positive for SARS-CoV-2 and age, sex and geographically matched SARS-CoV-2 negative controls with a follow-up at 3, 6, 12 and 24 months [40]. In a preliminary analysis of 6804 participants who completed the questionnaire at 3 months (3065 who tested positive and 3739 who tested negative), 66.5% and 53.3%, respectively, had any symptoms, and 30.3% versus 16.2% had three or more symptoms. The most common symptoms in both groups were headache and fatigue [41]. In the follow-up study at 6 months, which included 1658 SARS-CoV-2 positive and 1737 negative participants without evidence of reinfection (positive cohort) or new infection (negative cohort), most symptoms had declined, although tiredness persisted longer than other symptoms [42]. A single-center small US study also found that most symptoms had resolved by 6 months [43].

The incidence of long COVID in children and adults has declined with each new wave, which may reflect the change in viral variants and/or emergence of natural and/or vaccine immunity. For example, a prospective study from a single center in Turkey that queried 71 children infected during the Delta wave and 129 during the Omicron wave 12 weeks after diagnosis found that weakness (8.5% vs. 1.6%; *p* = 0.017), fatigue (22.5% vs. 8.5%; *p* = 0.009), anxiety (12.7% vs. 0.8%; *p* = 0.001) and gastrointestinal changes (12.7% vs. 4.7%, *p* = 0.050) were more common in patients infected during the Delta compared to Omicron wave [44]. A study in Thailand also found that persistent symptoms were more common among children infected with Delta compared with Omicron variants (36.3% vs. 23.9%, respectively) [45]. In a large retrospective study of adults and children from Sweden, Omicron was associated with a lower risk of long COVID in all subgroup analyses, including children, except the small subset of pediatric ICU-treated patients [46].

The decrease in incidence of long COVID with the emergence of Omicron and its subvariants is consistent with the various hypotheses proposed to cause this syndrome. These include a hyperimmune or aberrant immune response, the triggering of autoimmune responses, persistence of SARS-CoV-2 and/or reactivation of latent viruses. A longitudinal multi-omic study of patients from diagnosis to 2–3 months post-infection identified several factors associated with persistent symptoms, including initial disease severity and viral load (SARS-CoV-2 RNAemia), specific autoantibodies, EBV, and in some cases, CMV reactivation [47]. An excessive immune response may be a consequence of persistent antigenic stimulation from an ongoing reservoir of virus, for example, in the gut, whereas an impaired or dysregulated immune response might allow for viral persistence or the selection of autoantibodies. The decrease in disease severity associated with Omicron combined with the emergence of natural and/or vaccine immunity would be expected to mitigate against several of these factors and contribute to the decline in the incidence of long COVID. Consistent with this is the observation that long COVID is less common among vaccinated patients [48]. However, whether the immune response contributes to long COVID has been challenged by more recent studies. For example, no differences in antibody or T cell responses were found comparing healthcare workers with SARS-CoV-2 who did or did not have symptoms of long COVID [49].

## 6. What Makes the Acute and Post-Acute Response to SARS-CoV-2 Different in Children?

The pre-Omicron variants (Alpha and Delta) were associated with more severe acute disease and higher rates of post-acute sequelae compared to Omicron in both adults and children. However, across all waves, children consistently exhibited a milder clinical course than adults. The milder disease course cannot be attributed to differences in rates of infection, expression of ACE2 or TMPRSS2, viral loads or cross-reactivating antibodies to other coronaviruses [50,51,52] (Table 1). Rather, studies suggest that children mount a more vigorous innate response, which protects against severe disease. In a study comparing children and adults hospitalized with SARS-CoV-2 pre-Omicron and prior to the introduction of vaccines, we found that serum levels of IL-17A and IFN-γ were higher in children versus adults and correlated significantly and inversely with age [51]. The source of these cytokines was not likely the peripheral blood because adults had more robust spike-specific T cell responses; this led to the speculation that local innate responses may differ [51]. This hypothesis was supported by a subsequent study where the transcriptional profile of nasopharyngeal cells (bulk RNA sequencing) and quantification of mucosal cytokines and antibodies in nasopharyngeal swabs obtained from children or adults with SARS-CoV-2 were compared [50]. SARS-CoV-2 RNA copies, ACE2 and TMPRSS2 gene expression were similar in children and adults, but higher expression of genes associated with IFN signaling, NLRP3 inflammasome and other innate pathways were detected in the children compared to the adults. Consistent with the RNA data, protein levels of IFN-α2, IFN-γ, IP-10, IL-8 and IL-1β proteins were also higher in the nasal fluid of children versus adults, but anti-spike IgA and IgG were detected at similar levels in the nasal fluid of both groups. The notion that children mount a stronger local innate response was further supported by a study that used single-cell profiling of nasal, airway and blood samples from pediatric and adult patients. Interferon pathways were activated in SARS-CoV-2 uninfected healthy children compared to adults and this was further increased in those with SARS-CoV-2 infection. Conversely, adults with COVID-19 exhibited a greater peripheral blood cytotoxic T cell response.

In another study, clinically mild acute SARS-CoV-2 infection in children was associated with an increase in activated neutrophils but lower levels of circulating monocytes, dendritic cells and natural killer cells compared to blood samples obtained from adults with acute COVID-19. The authors speculated that the lower levels of these immune cells in the circulation may reflect their recruitment to mucosal sites to bolster the innate response [53]. These and other observations suggest that children may be primed at mucosal sites to respond to SARS-CoV-2, possibly because of more frequent respiratory infections. Another possible basis for the more robust pediatric innate response is increased expression of TLR2 by innate immune cells in children compared to adults, which may promote more rapid activation of interferon pathways [54].

This enhanced innate response may protect children from the vigorous systemic inflammatory response that has been shown to contribute to ARDS and other systemic disease manifestations observed in adults in the pre-Omicron era [54,55]. Importantly, this priming does not appear to interfere with the generation of SARS-CoV-2 antibody or memory T cell responses. A relatively large household study found that children had higher specific antibody levels which persisted for longer (96.2% versus 82.9% remained seropositive 11–12 months post-infection), despite being much more likely to be asymptomatic. Notably, symptomatic and asymptomatic infections induced similar antibody responses and there were no differences in the neutralization titers between adults and children [56,57]. A study on the immune cell profile of children compared to adults recovering from SARS-CoV-2 infection further supports the notion that immune activation contributes to the adverse outcomes. Adults had higher levels of activated and senescent cells, whereas children had higher T and B regulatory cell levels [58]. This may explain why children have less severe clinical manifestations attributable to immune activation and cytokine storm. A more recent study that took advantage of tonsillar and adenoidal tissue available from children with documented prior SARS-CoV-2 infection identified viral specific class-switched and somatically hypermutated B cells and expanded T cell clonotypes in the upper respiratory tract tissue [59]. These findings further support the notion that the innate response that protects children from severe disease does not prevent the development of a robust adaptive immune response.

## 7. Conclusions

Studies of COVID-19 in children have provided unique insights into how viral and host factors contribute to disease pathogenesis. For example, the decreased susceptibility of the Omicron variant spike proteins to TMPRSS2 cleavage resulted in greater dependence on entry via endocytosis rather than direct plasma membrane fusion and decreased syncytia formation. This impacted tissue and cell tropism, resulting in increased upper respiratory and less lung disease. The greater innate immune response to SARS-CoV-2 in children compared to adults, which was illustrated by several different studies comparing mucosal and systemic immune responses, presumably protected children from the more severe outcomes of COVID-19 that dominated the early waves of the pandemic. These findings underscore the importance of considering age-related host immune responses in exploring differences in disease manifestations and support the development of clinical studies to determine if treatment with exogenous sources of interferons could stimulate a stronger innate response and reduce disease severity in adults [60]. However, significant gaps in our understanding of COVID-19 in children remain, including the mechanisms underlying MIS-C and why it occurred more with early waves and predominantly in children. Similarly, the pathogenesis of the heterogenous condition designated as long COVID, which may be more common in adults, also requires further study. Studies continue to highlight the importance of COVID-19 vaccinations in children, as two recent pediatric studies showed protective effects of hybrid immunity (previous infection and vaccination) against SARS-CoV-2 infection [61,62].

## 8. Future Directions and Limitations

SARS-CoV-2 is now an endemic virus that will undoubtedly continue to evolve. While the majority of children have been relatively protected from severe disease, ongoing viral mutations may result in changes in cell and tissue tropism, clinical manifestations and susceptibility of the virus to innate and adaptive immune responses. For example, increased interferon resistance and a reduction in the ability of SARS-CoV-2 to activate immune responses have been observed with newer SARS-CoV-2 variants [63]. Moreover, the landscape may change. When SARS-CoV-2 first emerged, everyone, regardless of age, was immunologically naïve to the virus. However, as immunologic memory, albeit imperfect, elicited in response to natural infection and/or vaccines continues to expand, immunocompromised and unvaccinated persons including infants may become the most vulnerable. Therefore, we must continue to be vigilant and monitor the changes in the epidemiology including emerging viral variants and disease manifestations. It will also be important to comprehensively explore differences in innate and adaptive immune response across the full age spectrum. It is imperative that we promote vaccinations and prioritize research to prevent another viral pandemic. Ongoing and renewed research focused on why children responded differently and more favorably will continue to provide important insights into how to prepare and protect against new waves of SARS-CoV-2 or the next pandemic virus.

## Figures and Tables

**Table 1:** Age-associated host features and immune responses to SARS-CoV-2 that may contribute to disease severity.

Host Feature	Children versus Adults
Rates of infection and initial SARS-CoV-2 RNA copies	No differences
ACE-2 and TMPRSS2 expression	No differences
Innate responses nasal mucosa ↑expression IFN signaling, NLRP3 inflammasome transcripts	Increased in children
Systemic cytokine inflammatory response	Increased in adults
SARS-CoV-2 systemic neutralizing antibodies	No differences
Activated T cell responses	Increased in adults

## Abbreviations

ACE2	Angiotensin-converting enzyme-2
CDC	Center for Disease Control
cfDNA	Cell-free DNA
COVID-NET	US COVID-19-Associated Hospitalization Surveillance Network
ICU	intensive care unit
MIS-C	multisystem inflammatory syndrome in children
PASC	post-acute sequelae of SARS-CoV-2 infection
TMPRSS2	transmembrane protease serine 2
